# HIV self-testing in Rwanda: awareness and acceptability among male clinic attendees in Kigali, Rwanda: A cross-sectional survey

**DOI:** 10.1016/j.heliyon.2020.e03515

**Published:** 2020-03-07

**Authors:** Tafadzwa Dzinamarira, Claude Mambo Muvunyi, Collins Kamanzi, Tivani Phosa Mashamba-Thompson

**Affiliations:** a Department of Public Health Medicine, School of Nursing and Public Health, University of KwaZulu-Natal, Durban, 4001, South Africa; b College of Medicine and Health Sciences, University of Rwanda, Kigali, Rwanda; c CIHR Canadian HIV Trials Network, Vancouver, BC, Canada; d Department of Public Health, University of Limpopo, Polokwane, Limpopo Province, South Africa

**Keywords:** Health sciences, Public health, Infectious disease, Laboratory medicine, Social sciences, HIV self-Testing, Awareness, Acceptability, Men

## Abstract

**Background:**

The Rwandan Ministry of Health recently (in February 2017) recommended the use of HIV self-testing (HIVST) as an additional strategy for hard-to-reach populations such as men. However, the level of awareness and acceptability of this testing strategy among this population in Rwanda is not known. The main objective of this study is to assess the level of awareness and acceptability of HIVST among male clinic attendees in Kigali, Rwanda.

**Methods:**

A cross-sectional survey was employed to systematically sample and interview 579 male health-facility attendees over a seven-week period. We employed a pretested interviewer questionnaire to collect data. The chi-square test was used to determine associations between explanatory variables. Univariate binary logistic regression analysis was carried out to obtain preliminary insight into the unconditional association of each independent variable and dependent variables (awareness and acceptability). Multiple logistic regression was employed to determine explanatory variables associated with awareness or acceptability status while adjusting for other study variables. All statistical analyses were performed using Stata version 11.2.

**Results:**

Of the 579 men interviewed, only 21% were aware of HIVST, while 74% found it acceptable. Logistic regression analysis identified the following as factors significantly (p < 0.05) associated with HIVST awareness: having paid or received money for sex in the past month, health-seeking behavior, HIVST knowledge, HIVST attitude, and HIV risk perception. Factors associated with HIVST acceptability include the following: health-seeking behavior, HIVST knowledge, HIVST attitude, and condom use after taking drugs and alcohol.

**Conclusion:**

The findings reveal low awareness and high acceptability of HIVST among men in Rwanda. Our findings accentuate the need to promote awareness of HIVST as an important intervention for improving the uptake of HIV testing among men, a traditionally hard-to-reach population in Rwanda.

## 1 Introduction

Globally, men are considered a hard-to-reach and underserved population when it comes to HIV testing services [1]. Knowledge of one's HIV status is the first and most important step in the HIV care and treatment cascade [2,3,4,5]. In Rwanda, multiple interventions have been made to improve the uptake of HIV testing services (HTS), including voluntary counseling and testing (VCT) [6,7] and provider-initiated testing and counseling (PITC) [8,9]; however, the level of uptake remains low among men [10]. Since the World Health Organization (WHO) published the first guidelines on HIV self-testing (HIVST) in 2016 [11], a number of scholars have reported HIVST as a promising intervention capable of improving the proportion of men tested for HIV [12,13,14]. For instance, HIVST has been reported to be attractive to men mainly for its privacy and convenience [15,16], key barriers to male engagement in current facility-based HTS [17].

The decision to get tested for HIV should not compromise a patient's autonomy [18]. In 2010, the WHO highlighted the need for individuals to be given an option to decline HIV testing [19]. With VCT and PITC, men have raised concerns over confidentiality as a key barrier to HTS [20,21,22]. HIVST, on the other hand, has the potential to bridge this gap, as it allows individuals to collect their own sample and test in privacy [20,23,24,25]. HIVST has been reported to be reliable, safe, and accurate, with the potential to increase serostatus awareness rates and ultimately access to treatment for underserved populations [20,25]. HIVST has been successfully offered to men in facility-based settings [26,27] and community settings, such as workplaces [26], in Malawi, Zambia, and Zimbabwe. Several studies show that HIVST is an acceptable strategy in a variety of populations globally [21,23,28,29]. In addition, early studies in sub-Saharan Africa also reported high interest in HIVST among the general population, couples, high-risk populations, health care providers, and policy stakeholders [16,28,30]. A feasibility study conducted in Malawi [31] found that men preferred home-based self-test options for future testing. A systematic scoping review on men's perspectives toward HIVST in sub-Saharan Africa revealed that they perceived HIVST as an alternative, confidential, and convenient testing model and further recommended implementing community-level campaigns aimed at educating men about HIVST. However, there is limited evidence documenting the awareness and acceptability of HIVST among men in Rwanda.

Various reports and scholars have found mixed results in regard to meeting the global target to end AIDS by 2030 [32,33,34]. Early HIV diagnosis with ultimate linkage to treatment remains the foundation of meeting this target [35,36,37]. According to a recently concluded national HIV survey in Rwanda, 83.8% of adults living with HIV know their status, 97.5% of those individuals self-report being on antiretroviral therapy (ART), and 90.1% of those who report being on ART have viral load suppression [38]. By sex, 85.6% of HIV-positive women and 80.4% of HIV-positive men knew their HIV status [38]. This underscores the need to improve the first 90 among men. As of February 2017, it is recommended by the Rwanda Ministry of Health (MOH) to use HIVST kits as an additional strategy to reach out to people who have not yet been tested. Due to upcoming large-scale implementation of HIVST in Rwanda [39,40,41], it will be critical to understand men's awareness and acceptability of HIVST in order to design a successful program that reaches men, a population considered underserved in HTS.

## 2 Methods

### 2.1 Setting

Three health facilities, one from each district in Kigali, were purposely selected based on numbers of clinic attendees. Three facilities selected (University Teaching Hospital of Kigali (Nyarugenge District), Kibagabaga Hospital (Gasabo District), and Rwanda Military Hospital (Kicukiro District)) are tertiary facilities with large numbers of clinic attendees.

### 2.2 Recruitment of participants

We conducted the survey from July 1 to August 16, 2019. Trained interviewers systematically selected and interviewed male clinic attendees. We employed a systematic random sampling technique. Interviews lasted an average of forty minutes. Male clinic attendees who had visited the health facility as clients, accompanying or visiting relatives or for other reasons, were eligible for recruitment. We included participants who reported that they were unaware of their HIV status at the time of the study. We defined “unaware of HIV status” as never having taken an HIV test or having not had an HIV test in the past twelve months. Only men who were age 18 and older were eligible for this study. Signed written consent was obtained prior to any study procedures.

### 2.3 Data collection tool

The questionnaire was initially developed in English, then translated into Kinyarwanda by a public health specialist and then translated back into English by another public health specialist to ensure translation accuracy. We piloted both versions (English and Kinyarwanda) on a sample of 15 men before the data collection. A few translation discrepancies were identified and resolved by investigators, in consultation with the translators. The questionnaire addressed the sociodemographic characteristics of the interviewee (sex, age, education, place of residence, marital status, and occupation), sexual health, HIV risk perception, outcome variables (awareness of HIVST, acceptability of HIVST), and knowledge and attitudes toward HIVST. HIVST was introduced to respondents in text: “As of 20 February 2017, it is recommended by the MOH to use HIVST kits as an additional strategy to reach out to people who are not yet tested, and there are plans for a national roll-out in 2019.”

#### 2.3.1 Outcome measures

This study assessed proportion of HIVST awareness and acceptability among men in Kigali, Rwanda.

##### 2.3.1.1 Awareness of HIVST

Awareness of HIVST was assessed by asking: “Do you know what HIVST is?” A binary yes (aware) or no (unaware) response was captured.

##### 2.3.1.2 Acceptability of HIVST

Acceptability of HIVST was assessed by asking: “Do you agree with this recommendation for HIVST?” Responses were initially captured in a five-point Likert scale: strongly disagree, disagree, neutral, agree, and strongly agree. The data were combined into two nominal categories: acceptable (strongly agree, agree) and unacceptable (neutral, disagree, strongly disagree) to allow for further analysis using the chi-square test.

#### 2.3.2 Exploratory variables

##### 2.3.2.1 Knowledge of HIVST

To assess HIV testing knowledge, participants were asked eight statements pertaining to test kit availability in Rwanda and general HIV self-testing methodology. The mean (SD) HIV testing knowledge score was 22 (4.52). The sum of responses were therefore rated as follows: ≤2 was “poor” and ≥3 was “good.” The scale had an acceptable level of internal reliability as determined by a Cronbach's alpha of 0.7016 with an average interim covariance of 0.3974.

##### 2.3.2.2 Attitude toward HIVST

A questionnaire was employed to evaluate the level of attitude among the study participants, which consisted of six statements that reflected general impressions of HIVST, willingness to accept results from HIVST, and post-test perceived care and treatment. Responses were initially captured on a five-point Likert scale and ultimately combined into two nominal categories, positive and negative, to allow for further analysis using the chi-square test. The mean (SD) attitude score was 23 (1.70). The sum of responses was therefore scaled as follows: ≤22 was “negative” and ≥23 was “positive.” The scale had a good level of internal reliability as determined by a Cronbach's alpha of 0.8018 with an average interim covariance of 0.6018.

### 2.4 Data management and analysis

Completed questionnaires were sent directly to the main survey server whenever transmission was possible. Thereafter the responses from all the questionnaires were captured using Excel for validation. The data set was exported to STATA software version 11.2 (STATA Corp., Texas, USA) for cleaning and coding to detect out-of-range and missing values. This was done using frequency tables and cross-tabulation for categorical variables and for continuous variables, and the data were summarized using means and standard deviations.

A univariate binary logistic regression analysis was carried out to obtain preliminary insight into the unconditional association of each independent variable and dependent variables (awareness and acceptability), and statistical significance was set at p < 0.05. Furthermore, to assess the net effect of each independent variable on dependent variables, a multivariable binary logistic regression was carried out by controlling for the effects of all other intervening variables so as to not to miss out some significant variables. This was done by including all variables with a *p* value less than 0.05 from the univariate analysis in the multivariate logistic regression model. Statistical significance was set at p = 0.05. In addition, odds ratios and 95% CI estimates were used to examine the associations. In the multivariable logistic regression, insignificant variables were subsequently removed from the models in backward stepwise fashion if their p > 0.05 until a final model was obtained that included the significant variables that influence the awareness and acceptability of HIVST.

#### 2.4.1 Ethics

This study was ethically reviewed and approved by four institutional review boards: the Rwanda National Ethics Committee (approval number: 332/RNEC/201), the University Teaching Hospital of Kigali Ethics Committee (approval number: EC/CHUK/0111/2019), the Rwanda Military Hospital Institutional Review Board (approval number: RMH IRB/036/2019), and the University of KwaZulu Natal Biomedical Research Ethics Committee (approval number: BE/280/19). Study participants were provided with an information sheet explaining the objectives of the study, and all participants signed paper informed consent forms prior to participation.

## 3 Results

### 3.1 Characteristics of study participants

#### 3.1.1 Demographic characteristics

The mean age of men who participated in this study was 28 years (standard deviation: 8). Most (53%) of the study participants had secondary education, while 38% were unemployed. The majority (60%) of the participants resided in the Gasabo District. More information on the demographic characteristics of the participants is presented in Table 1.

**Table 1:** Participant demographics, sexual health, and health-seeking behavior characteristics.

Variable	Mean	SD	Frequency n (%)
Age			

18-53	28	7.25	

Age at marriage			

18-37	27	3.62	

Marital status			

Married			160 (28)
Other			26 (4)
Single			393 (68)

Education level			

University			97 (17)
Secondary			309 (53)
Primary			156 (27)
Do not know			17 (3)

Income level			

Unemployed			220 (38)
Self-employed			210 (36)
Professional			149 (26)

Location			

Gasabo			347 (60)
Nyarugenge			119 (21)
Kicukiro			113 (20)

Sexual preference			

Heterosexual			558 (96)
Homosexual			10 (2)
Bisexual			11 (2)

Age at first sex			

13-30	21	3.62	

Number of sexual partners			

1-20	3.66	4.59	

Number of sexual partners in the last 12 months			

0-6	1.37	1.49	

Number of sexual partners in marriage			

0-10	1.49	3.72	

Sexual encounter			

Yes			435 (75)
No			144 (25)

Circumcised			

Yes			466 (80)
No			113 (20)

Paid/received money for sex			

Yes			46 (11)
No			389 (89)

Ever had STI			

Yes			27 (6)
No			408 (94)

Used condom at first sex			

Yes			204 (47)
No			231 (53)

How often do you and your partner use a condom?			

Never			144 (33)
Rarely			93 (21)
Sometimes			122 (28)
Always			76 (18)

Used condom in the last 12 months			

Never			200 (46)
Rarely			88 (20)
Sometimes			96 (22)
Always			51 (12)

HIV risk perception			

Very high			101 (17)
Fairly high			142 (25)
Moderate			131 (23)
Low			205 (35)

Reason for visiting hospital today			

Accompanying a relative			181 (31)
Accompanying a spouse			74 (13)
Visiting a relative			50 (9)
Seeking health services			274 (47)

How often did you get sick in the last 3 months?			

Never			302 (52)
1–3 times			195 (34)
4–5 times			50 (8)
More than 5 times			32 (6)

The last 3 times you were sick, what did you do first?			

Nothing			253 (44)
Consulted a qualified medical practitioner			259 (44)
Consulted a community health worker			15 (3)
Consulted an over-the-counter pharmacist			41 (7)
Consulted family members			11 (2)

When did you last visit a health facility for an ordinary check-up?			

Never			254 (44)
1–3 months ago			153 (26)
4–6 months ago			44 (8)
6–12 months ago			47 (8)
More than a year ago			81 (14)

#### 3.1.2 Sexual health characteristics

Of the participants, 76% had a sexual encounter before, and the average age at their sexual debut was 21 years. The median number of sexual partners in their lifetime and in the last 12 months was 4 and 2, respectively. Only 11% and 5% paid and/or received payment for sex in their lifetime and in the last 12 months, respectively. Most men (80%) were circumcised, and only 5% reported having had a sexually transmitted infection (STI) before. More than half (57%) of the men had their sexual partner share their HIV status with them. Less than half (43%) reported that they perceived themselves to be at risk of HIV infection. More information on the sexual health characteristics of the participants is presented in Table 1.

#### 3.1.3 Health-seeking behavior characteristics

Of the participants, 44% had visited a health facility or professional medical practitioner the last time they were sick. More than half (56%) reported having visited a health facility for an ordinary check-up in the past year. More information on the health-seeking behavior characteristics of the participants is presented in Table 1.

### 3.2 Awareness of HIVST

**Figure 1:**
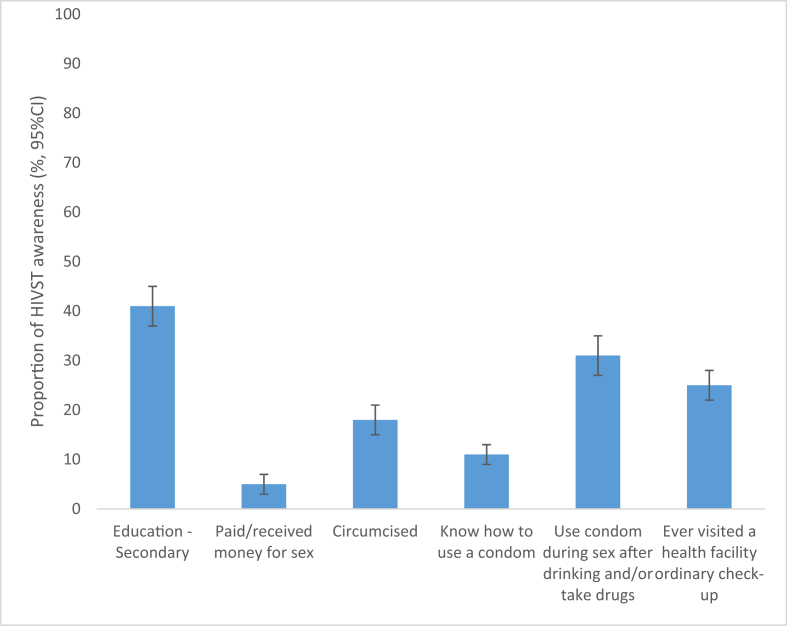
Proportion of HIVST awareness (%, 95% CI) reported by men in Kigali, Rwanda, *Error bars represent 95% confidence intervals, which indicate the interval within which the true population parameter is expected to fall 95% of the time.*

Of the 579 men interviewed, 21% were aware of HIVST. There was an association between education level and awareness status (p < 0.001): 41% among those with a university education were aware of HIVST compared to 20% and 10% among those with secondary or primary education, respectively. There was an association between having ever paid and/or received payment for sex and HIVST awareness status (p = 0.038): 5% among those having paid and/or received payment for sex were aware of HIVST compared to 22% among those having never paid. There was an association between circumcision status and HIVST awareness status (p = 0.003): 18% among those circumcised were aware of HIVST compared to 22% among those not circumcised. More details are presented in Figure 1.

### 3.3 Factors associated with HIVST awareness

**Figure 2:**
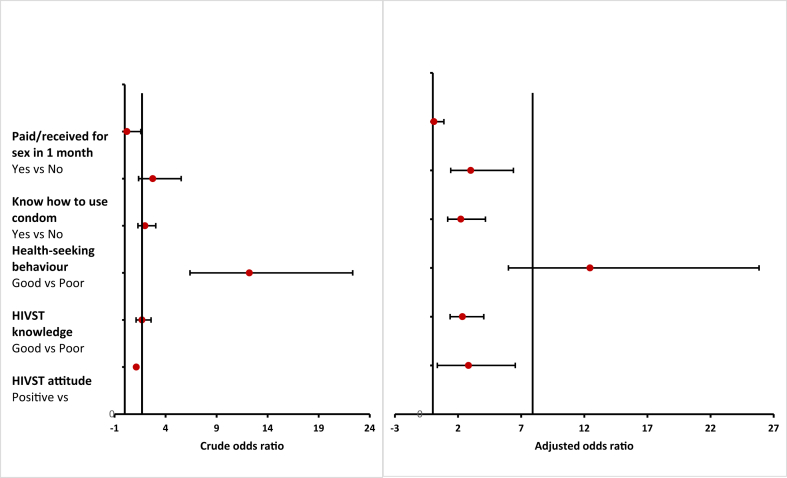
Factors associated with awareness of HIV self-testing among men in Kigali, Rwanda.

In the bivariate model, education level, circumcision, having paid or received money for sex in the last month, knowing how to use a condom, HIV risk perception, health-seeking behavior, HIVST knowledge, and HIVST attitude were significantly associated with HIVST awareness (p < 0.05). More details are presented in Table 2. These eight variables were included in the multivariate logistic regression model. In the multivariate logistic regression, the following variables were shown to be significantly associated with HIVST awareness: having paid or received money for sex in the past month, health-seeking behavior, HIV risk perception, knowledge of HIVST, attitude toward HIVST (p < 0.05). More details are presented in Table 2 (see Figure 2).

**Table 2:** Univariate and multivariate analysis results for factors associated with HIVST awareness and acceptability among men in Kigali, Rwanda.

Variables	HIVST Awareness				HIVST Acceptability			
	Odds ratio (95% CI)	*p* value	Adjusted odds ratios (95% CI)	*p* value	Odds ratio (95% CI)	*p* value	Adjusted odds ratios (95% CI)	*p* value
Age in years	0.98 (0.95–1.00)	0.095	-	-	1.05 (1.02–1.08)	**0.02**	-	-

Marital status								

Single	Reference	Reference	-	-	Reference	Reference	-	-
Married	0.86 (0.54–1.36)	0.15	-	-	1.06 (0.70–1.61)	0.63	-	-
Others	0.29 (0.07–1.28)		-	-	1.59 (0.59–4.33)		-	-

Education level								

Do not know	Reference	Reference						
University	3.14 (0.85–11.65)	**0.000**			3.22 (1.11–9.36)	0.11	-	-
Secondary	1.12 (0.31–4.04)				2.19 (0.82–5.88)		-	-
Primary	0.57 (0.15–2.19)				2.86 (1.03–7.94)		-	-

Income level								

Unemployed	Reference	Reference	-	-	Reference	Reference	-	-
Self-employed	0.99 (0.60–1.64)	0.41	-	-	2.97 (1.89–4.68)	**0.000**	-	-
Professional	0.75 (0.46–1.20)		-	-	1.74 (1.09–2.75)			

Location								

Gasabo	Reference	Reference	-	-	Reference	Reference	-	-
Nyarugenge	1.18 (0.71–1.96)	0.81			0.32 (0.20–0.50)	**0.000**	-	-
Kicukiro	1.03 (0.61–1.75)				0.49 (0.31–0.79)			

Sex preference								

Heterosexual	Reference	Reference	-	-	1.86 (0.52–6.69)	0.51	-	-
Homosexual	2.39 (0.29–19.04)	0.38	-	-	Reference	Reference	-	-
Bisexual	0.89 (0.05–16.59)				1.17 (0.20–6.80)			

Age at first sex								

Early (<18)	Reference	Reference			Reference	Reference	-	-
Late (>18)	0.86 (0.66–1.12)	0.260			1.45 (1.13–1.86)	**0.003**	-	-

Sexual encounter								

No	Reference	Reference	-	-	Reference	Reference	-	-
Yes	1.23 (0.76–1.99)	0.39	-	-	1.75 (1.16–2.62)	**0.001**	-	-

Circumcised								

No	Reference	Reference	-	-	Reference	Reference	-	-
Yes	3.57 (1.75–7.29)	**0.001**	-	-	1.10 (0.69–1.74)	0.68	-	-

Paid/received for sex in the last month								

No	Reference	Reference	Reference	Reference	Reference	Reference	-	-
Yes	0.21 (0.03–1.58)	**0.049**	0.09 (0.007–0.88)	0.038	0.48 (0.78–1.26)	0.15	-	-

Ever had STI								

No	Reference	Reference	-	-	Reference	Reference	-	-
Yes	0.83 (0.30–2.24)	0.70	-	-	4.17 (0.97–17.89)	**0.02**	-	-

Partner shared HIV status								

Never	Reference	Reference			Reference	Reference	-	-
Rarely	1.04 (0.52–2.06)	0.06			0.49 (0.28–8.70)	**0.040**	-	-
Sometimes	2.34 (1.21–4.50)				1.42 (0.65–3.09)		-	-
Always	1.64 (0.87–3.07)				1.12 (0.58–2.17)		-	-

Know how to use condom								

No	Reference	Reference	Reference	Reference	Reference	Reference	-	-
Yes	2.75 (1.36–5.53)	**0.0019**	3.01 (1.42–6.39)	0.004	1.27 (0.75–2.12)	0.38	-	-

Received condoms in the last 12 months								

Never	Reference	Reference	-	-	Reference	Reference	-	-
Rarely			-	-	1.15 (0.63–2.09)	0.09	-	-
Sometimes			-	-	1.62 (0.89–2.94)		-	-
Always			-	-	0.62 (0.32–1.20)		-	-

Condom use after drugs and alcohol								

Never	Reference	Reference	-	-	Reference	Reference	Reference	Reference
Rarely	1.003 (0.51–1.96)	0.86	-	-	0.15 (0.06–0.35)	**0.000**	0.25 (0.07–0.96)	0.044
Sometimes	1.02 (0.43–2.41)		-	-	0.29 (0.14–0.60)		0.44 (0.13–1.44)	
Always	1.75 (0.53–5.79)		-	-	0.48 (0.22–1.03)		0.50 (0.17–1.47)	

HIV risk perception								

Low	Reference	Reference	Reference	Reference	Reference	Reference	-	-
High	1.13 (1.04–1.23)	**0.0048**	2.83 (0.36–6.53)	0.015	2 (1.15–3.50)	**0.000**	-	-

Health-seeking behavior								

Poor	Reference	Reference	Reference	Reference	Reference	Reference	Reference	Reference
Good	1.98 (1.29–3.04)	**0.0014**	2.22 (1.18–4.17)	0.0013	0.47 (0.32–0.70)	**0.000**	0.48 (0.26–0.88)	0.02

HIVST knowledge								

Poor	Reference	Reference	Reference	Reference	Reference	Reference	Reference	Reference
Good	12.21 (6.39–22.33)	**0.00001**	12.45 (5.99–25.84)	0.000	2.09 (1.44–3.05)	**0.0001**	2.95 (1.66–5.23)	0.000

HIVST attitude								

Negative	Reference	Reference	Reference	Reference	Reference	Reference	Reference	Reference
Positive	1.69 (1.11–2.58)	**0.01**	2.35 (1.37–4.04)	0.002	4.12 (2.78–6.10)	**0.000**	2.55 (1.39–4.65)	0.002

### 3.4 Acceptability of HIVST

**Figure 3:**
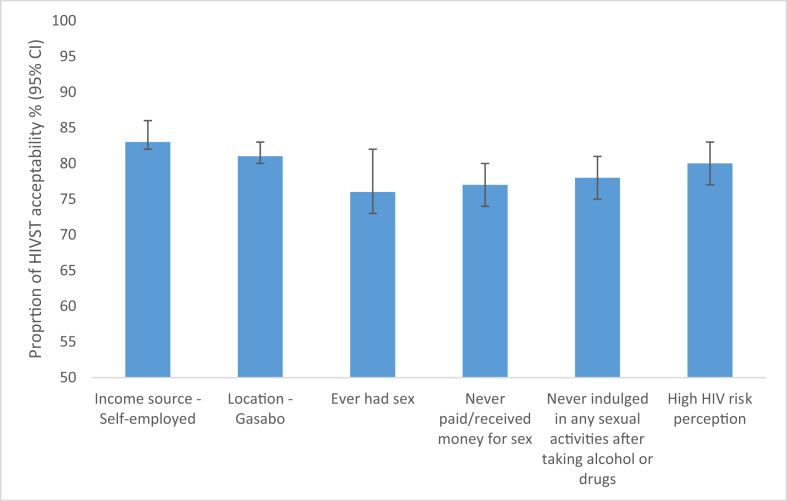
Proportion of HIVST acceptability (%, 95% CI) reported by men in Kigali, Rwanda.

Of the 579 men interviewed, 74% found HIVST acceptable. There was an association between income source and HIVST acceptability (p < 0.001): 83% among those self-employed found HIVST acceptable compared to 75% and 62% among professionals and the unemployed, respectively. There was an association between location and HIVST acceptability (p < 0.001): 81% among those reporting residing in the Gasabo district found HIVST acceptable compared to 66% and 57% among residents of Kicukiro and Nyarugenge, respectively. There was an association between ever having had a sexual encounter and HIVST acceptability (p = 0.004): 76% among those who reported having had a sexual encounter found HIVST acceptable compared to 64% among those who reported never having had a sexual encounter. More details are presented in Figure 3.

Error bars represent 95% confidence intervals, which indicate the interval within which the true population parameter is expected to fall 95% of the time.

### 3.5 Factors associated with HIVST acceptability

**Figure 4:**
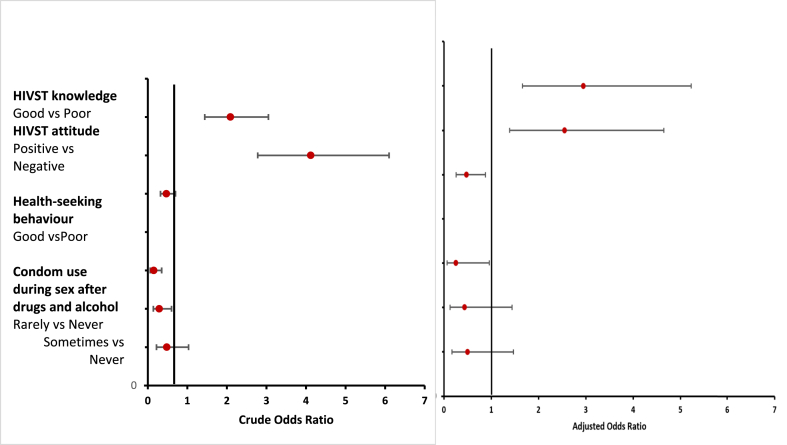
Factors associated with the acceptability of HIVST among men in Kigali, Rwanda.

In the bivariate model, the following variables were shown to be significantly associated with acceptability (p < 0.05) (Table 2): age, income level, location of residence, ever had sex, age at first sex, ever had an STI, partner shared HIV status, condom use after drugs and alcohol, received a condom in the last 12 months, health-seeking behavior, HIV risk perception, HIVST knowledge, and HIVST attitude. In the multivariate logistic regression, the following variables were shown to be significantly associated with HIVST awareness (p < 0.05): HIVST knowledge, HIVST attitude, health-seeking behavior, and condom use after taking drugs and alcohol (Figure 4).

## 4 Discussion

The main aim of this study was to explore the awareness and acceptability of HIVST among men in Rwanda. In this study, only 21% of the men were aware of HIVST, while 74% found it acceptable. A key focus set forth in the Rwanda 2019–2024 Fourth Health Sector Strategic Plan [42] is improving uptake of HTS among men as an important step in achieving the UNAIDS 90-90-90 target of 2020 [43]. HIVST has been reported to have potential to bridge this gap [44,45]. In the current study, the findings revealed that knowledge of how to use condom, health-seeking behavior, HIVST knowledge, HIVST attitude, and HIV risk perception were found to be factors associated with HIVST awareness. The findings of the present study revealed health-seeking behavior, HIVST knowledge, and HIVST attitude to be associated with HIVST acceptability. The findings corroborate earlier calls to address poor health-seeking behavior among men [46]. Interventions to address men's health-seeking behavior have been recommended earlier, in the WHO country cooperation strategic agenda for Rwanda (2014–2018) [47]. In response, a number of health policies and systems have been employed to facilitate universal health coverage for all [47,48]. Our findings show a need to further strengthen these efforts.

The findings of the current study revealed poor awareness (21%) and high acceptability (74%) among men in Kigali, Rwanda. Other studies that have been carried out in different other settings have given higher rates than the current study. For instance, in Australia, 41.9% of bisexual men were aware of HIVST intervention [49]. In a different study conducted in eThekwini district, KwaZulu-Natal Province, South Africa, in 2018, the findings established a higher level of awareness of HIVST (69.9%) [50]. The difference from the current study findings may be attributed to the fact that in the study conducted in South Africa, only 30.1% of the respondents were male, while 68.8% were female [50]. Higher rates of awareness have been reported in Kenya and South Africa [51,52]. This may be because HIVST is a relatively new intervention in Rwanda, currently at pilot stage [5,10,39]. In the current study, the findings revealed that knowledge of how to use condom, health-seeking behavior, HIVST knowledge, HIVST attitude, and HIV risk perception were found to be factors associated with HIVST awareness. This corroborates the findings of a study that was carried out by Izizag et al. (2018) in the Democratic Republic of Congo [53]. In their study, majority (57%) of the participants were male [53]. Similar to findings from the current study, their findings also noted that the awareness of HIVST was associated with knowledge of HIVST [53]. The findings of the present study noted that the rate of acceptability of HIVST was 74%. In South Africa, 75% of students reported HIVST to be acceptable [54]. In that study, 3,662 students took part, with 43.4% being male [54]. Similarly, high acceptability rates were reported in Kenya [55]. The findings of the current study are also in tandem with findings from a systematic review conducted by Krause *et al.* (2013) [30] and more recent literature reviews by Figueroa *et al.* (2015) [25]. Sharma *et al*., (2015) [36]. Acceptability rates in sub-Saharan Africa have been reported as ranging from 22% to 94% [56,57], with a gendered pattern of men benefitting more from HIVST than women [52,58]. Acceptability rates of HIVST in studies with men only were higher (>70–94%) when compared to studies with men and women (22–64%) [51,52,58,59]. The findings of the present study indicated that some of the key factors linked to acceptability are health-seeking behavior and HIVST knowledge. Izizag *et al.* (2018) also reported similar findings [53].

HIVST may be attractive to men in Rwanda. The current study findings demonstrate a need for strengthening HIVST awareness among men to improve uptake. However, before effective implementation, more factors, like access to HIVST, cost, false reassurance from the test, missed early infections during the window period, and limited counseling, as well as linkage to care options, ought to be taken into consideration [60,61]. Policy makers need to ensure that various programs are designed to make HIVST more appealing to those who previously never took advantage of HTS for whatever reasons. Further research incorporating key stakeholders is required to identify demand creation strategies that can lead to better HIVST uptake among men. Policy guidance should also inform acceptance of HIVST, taking into consideration diversity within the target populations, options for and the reliability of testing techniques, the components of the test kits that are used, the clarity of user instructions in relation to varying literacy levels, disposal of the test kit, counseling techniques, and linkage to care options. At the middle and lower stakeholder levels, the capacity building of providers to be part of the team that creates awareness can lead to better awareness and acceptability levels. Various studies have indicated that the evidence on the usability of HIVST kits generally remains limited across the globe, including major gaps in sub-Saharan Africa [15,56,57,62,63,64].

To fully understand awareness and acceptability levels among men in Kigali, a household-based cross-sectional study would have been ideal. A limitation of this study is that the sample was drawn from male clinic attendees who were already receiving health services and may have been more willing to get tested at a facility. HIVST can be a vehicle for HIV testing among men who do not want to attend health facilities; the current study did not reach this critical population who may have inherently different perspectives about HIVST use. However, we believe we have produced valuable findings, as over half the participants were males accompanying or visiting someone at the clinic; they could represent the population of men who are hard to reach with facility-based HTS.

The study achieved its aim and revealed a gap in HIVST awareness among men in Kigali, Rwanda. Policy makers ought to adopt various measures to facilitate its implementation and scale-up. More implementation research, as well as interventions, are needed with the aim of improving the acceptability of HIVST among diverse study populations. Although the study found low awareness of HIVST among men in Rwanda, the findings indicate that offering HIVST as an option for HIV testing is highly acceptable to men. As HIVST becomes increasingly available and awareness is raised among men about its benefits, HIVST has strong potential as a tool for helping more men know their HIV status in Rwanda.

## 5 Conclusion

The findings reveal low awareness and high acceptability of HIVST among men in Rwanda. Our findings accentuate the need to promote awareness of HIVST as an important intervention for scaling uptake of HIV testing among men, a traditionally hard-to-reach population in Rwanda.

## Declarations

### Author contribution statement

T. Dzinamarira: Conceived and designed the experiments; Performed the experiments; Analyzed and interpreted the data; Wrote the paper.

C. Muvunyi: Conceived and designed the experiments; Analyzed and interpreted the data.

C. Kamanzi: Performed the experiments; Analyzed and interpreted the data.

T. Mashamba-Thompson: Conceived and designed the experiments; Analyzed and interpreted the data; Wrote the paper.

### Funding statement

This work was supported by The University of KwaZulu-Natal, College of Health Sciences PhD Scholarship and the CIHR Canadian HIV Trials Network (CTN 222). T. Dzinamarira was supported by The University of KwaZulu-Natal, College of Health Sciences PhD Scholarship. T. Mashamba-Thompson was supported by CTN Postdoctoral Fellowship Award.

### Competing interest statement

The authors declare no conflict of interest.

### Additional information

No additional information is available for this paper.
